# Clinical Characteristics and Outcomes of Elderly Patients Undergoing Primary Percutaneous Coronary Intervention: An Observational Cohort Study

**DOI:** 10.5334/gh.1383

**Published:** 2024-12-30

**Authors:** Ahmed Hassan, Amr Yosry Emam, Mohammed Thabet, Ahmed Osman, Khaled Ahmed Shams, Mina Samir Labib, Ahmed Elguindy

**Affiliations:** 1 Cairo University, Egypt; 2 Adult Cardiology Department, Aswan Heart Centre, Magdi Yacoub Foundation, Aswan, Egypt; 3 Cardiology Department, Cairo University, Cairo, Egypt; 4 Department of Cardiovascular Medicine, Kasr Alainy Faculty of Medicine, Cairo University, Egypt; 5 Cardiology Department, Helwan University, Helwan, Egypt; 6 Aswan Heart Centre, Magdi Yacoub Heart Foundation, Egypt

**Keywords:** primary percutaneous coronary intervention, elderly, septuagenarians, octogenarians

## Abstract

**Background:**

The global trend of population aging has resulted in more frequent cardiovascular disease among seniors. Primary percutaneous coronary intervention (pPCI) is the standard of care for ST-elevation myocardial infarction (STEMI) without an upper age limit. Nevertheless, the outcomes are variable among studies, and data on pPCI outcomes in the elderly in Africa is scarce. Thus, we attempted to gain better insight into the outcomes of primary PCI in this age group from a single center in upper Egypt.

**Objective:**

To study the patient characteristics and in-hospital outcomes of pPCI in elderly patients presenting with STEMI in a tertiary cardiac center in upper Egypt.

**Methods and results:**

This observational cohort study was based on data from the pPCI registry in a tertiary cardiac center in upper Egypt, which included 3,627 consecutive patients who underwent pPCI between January 2014 and June 2023. The elderly were defined as those aged 70 years or older and represented 15.9% (575 patients) of the entire cohort, of whom 103 (2.8%) were octogenarians. Clinical characteristics, procedural details, and in-hospital outcomes were compared between the age groups. The elderly had a significant trend of being female and hypertensive, and having chronic kidney disease (CKD), worse Killip class, more frequent severe non-culprit vessel lesions, and left main trunk involvement. The in-hospital mortality was significantly higher than that of younger patients (14.1 vs. 4%, p = <0.001), with higher mortality in octogenarians (23.3%). Killip class ≥II was independently associated with increased hospital mortality in all study age groups. Contrast-induced nephropathy and TIMI major bleeding were also significantly higher.

**Conclusion:**

Compared to younger patients, elderly patients undergoing pPCI had a higher prevalence of hypertension and CKD and were more likely to have a worse Killip class. The radial approach was utilized less often in the elderly group. In-hospital complications and mortality, particularly among the octogenarians, were significantly higher than in younger patients.

## Introduction

Coronary artery disease (CAD) is a major cause of illness and death (1). The risk of mortality due to coronary atherosclerosis increases with age. Elderly patients with STEMI may present atypically, which can lead to misdiagnosis or treatment delay. Implementing the same revascularization strategy in older patients may be challenging due to comorbidities and healthcare professionals’ risk concerns (2).

Recently, there has been a notable rise in pPCI-capable hospitals in low- and middle-income countries with a better referral system. As a result, there has been a rise in the percentage of elderly patients referred for pPCI. The guidelines emphasize that an early invasive strategy for STEMI is the gold standard regardless of age (3). However, the management of STEMI in the elderly population is still challenging, and improved strategies are needed for early diagnosis and treatment, including managing associated co-morbidities and complications. We aimed to study the characteristics of older patients presenting with STEMI and define pPCI outcomes in this cohort.

## Methods

### Setting and design

The present study is part of a tertiary care center’s ongoing primary PCI registry, which started in 2014. The present study aims to assess the in-hospital outcome of pPCI and its predictors among the elderly.

### Study population

The study enrolled 3,627 patients who underwent pPCI between January 2014 and June 2023. They were categorized into two age groups: those under 70 and those over 70. The latter group was further divided into two subgroups: Septuagenarians and Octogenarians. Septuagenarian patients were defined as those aged 70 to 79, while Octogenarian patients were defined as those aged 80 years and above.

### Endpoints and measurements

The primary endpoint was in-hospital mortality. The secondary endpoints were the incidence of in-hospital complications (contrast-induced nephropathy (CIN), stroke, TIMI major bleeding, and in-hospital stent thrombosis). We analyzed various demographic and clinical factors, including age, gender, cardiovascular risk factors, clinical history of previous revascularization, comorbidities, ischemic time, Killip class, and basic angiographic data.

### Statistical analysis

Patient characteristics were presented as percentages for categorical variables and mean ± standard deviation (SD) for continuous variables. We utilized Pearson’s chi-square test to compare the outcomes of pPCI in elderly and younger patients for categorical variables. We used the two-sample t-test or Wilcoxon rank sum test to compare continuous variables between the study groups. Estimates of the odds ratios (OR) and 95% confidence intervals (CI) were obtained using univariate and multivariable logistic regression analyses to determine the predictors of outcome. The statistical significance was defined as a value of p < 0.05. Version 26 of SPSS was utilized for statistical analysis in this study.

## Results

From January 2014 to June 2023, 3,627 patients underwent pPCI. Among them, 3,052 patients (84.1%) were under 70, while 575 patients (15.9%) were over 70. The Septuagenarians were 472 patients (13%), while the Octogenarians were 103 patients (2.8%).

### Patient characteristics

Comparisons of demographic, clinical, and angiographic variables between elderly (≥70 years) and younger (<70 years) populations are shown in Table 1A. Compared to the younger cohort, the elderly group had more women (34.3% vs. 18.3%, p < 0.001), and was more likely to be hypertensive (57.7% vs. 40.9%, p < 0.001) and to have chronic kidney disease (37.6 % vs. 8.2%, p < 0.001). On the other hand, the younger cohort was more likely to be smokers (64.4% vs. 40.5%, p < 0.001). Upon presentation, the elderly were more likely to present with Killip class ≥2 (28% vs. 15.3%, p < 0.001) and cardiogenic shock (11.7% vs. 5.1%, p < 0.001), and to have longer ischemic time (6.4 hrs. vs. 5.8 hrs., p 0.007). Regarding the angiographic variables, left main coronary artery (LMCA) lesions (5.7% vs. 3.1%, p < 0.001) and right coronary artery (RCA) lesions (28% vs. 15.3%, p < 0.001) were more common in the elderly cohort.

**Table 1A:** Comparisons of demographic, clinical, and angiographic variables between elderly and the younger populations.


VARIABLES	AGE GROUP ≥70 YEARS (N = 575)	AGE GROUP <70 YEARS (N = 3052)	*P* VALUE

Demographic and clinical variables			

Women	197 (34.3%)	559 (18.3%)	**<0.001**

Hypertension	332 (57.7%)	1248 (40.9%)	**<0.001**

Diabetes mellitus	277 (48.2%)	1352 (44.3%)	0.171

Current smoker	233 (40.5%)	1964 (64.4%)	**<0.001**

History of previous revascularization	33 (5.7%)	198 (6.5%)	0.285

Chronic kidney disease	216 (37.6 %)	250 (8.2%)	**<0.001**

Killip class ≥II at presentation	161 (28%)	468 (15.3%)	**<0.001**

Cardiogenic shock at presentation	67 (11.7%)	155 (5.1%)	**<0.001**

Total Ischemic time (hours) Median, interquartile range	6.4 (4.0–10)	5.8 (3.7–9.2)	0.007

Radial access	349 (60.7%)	2143 (70.2%)	**<0.001**

Angiographic variables			

Associated severe LM disease	33 (5.7%)	95 (3.1%)	0.001

Proximal LAD culprit	235 (40.9%)	1158 (37.9%)	0.77

Non proximal LAD culprit	135 (23.5%)	802 (26.3%)	0.057

LCX lesion culprit	82 (14.3%)	516 (16.9%)	0.204

RCA lesion culprit	199 (34.6%)	851 (27.9%)	<0.001

Ramus lesion culprit	6 (1%)	22 (0.7%)	0.431

Large thrombus burden	92 (16.0%)	578 (18.9%)	0.194

Final TIMI flow 0 or 1	6 (1.0%)	27 (0.9%)	0.694

Other non-culprit vessel severe lesions	232 (40.3%)	936 (30.7%)	**<0.001**


Demographic, clinical, and angiographic characteristics of octogenarians (≥80 years) and Septuagenarians (70–79 years) are shown in Table 1B. Compared to the Septuagenarian cohort, octogenarians were more likely to have chronic kidney disease (55.34 % vs. 34.11%, p < 0.001).

**Table 1B:** Comparisons of demographic, clinical, and angiographic variables between the Octogenarians and Septuagenarians subgroups.


VARIABLES	OCTOGENARIANS (≥80 YEARS) (N = 103)	SEPTUAGENARIANS (AGE OF 70–79 YEARS) (N = 472)	*P* VALUE

Demographic and clinical variables			

Women	32 (31.1%)	165 (34.9%)	0.493

Hypertension	61 (59.2%)	274 (58.05%)	0.857

Diabetes mellitus	48 (46.6%)	232 (49.15%)	0.790

Current smoker	39 (37.9%)	196 (41.5%)	0.516

History of previous revascularization	10 (9.7%)	64 (13.6%)	0.602

Chronic kidney disease	57 (55.34 %)	161 (34.11%)	**<0.001**

Killip class ≥II at presentation	33 (32.03%)	128 (27.11%)	0.334

Total-Ischemic time (hours) Median, interquartile range	7 (4.5–10.6)	6 (4.0–9.6)	0.154

Angiographic variables			

Severe LM disease	10 (9.7%)	23 (4.9%)	0.072

Proximal LAD culprit	38 (36.9%)	197 (41.7%)	0.582

Non proximal LAD culprit	22 (21.4%)	113 (23.9%)	0.847

LCX lesion culprit	17 (16.5%)	65 (13.8%)	0.759

RCA lesion culprit	39 (37.9%)	160 (33.9%)	0.712

Ramus lesion culprit	1 (1%)	5 (1.1%)	0.755

Large thrombus burden	17 (16.5%)	75 (15.9%)	0.679

Final TIMI flow 0 or 1	2 (1.9%)	4 (0.8%)	0.077


### Outcomes

#### Primary outcome

**Figure 1:**
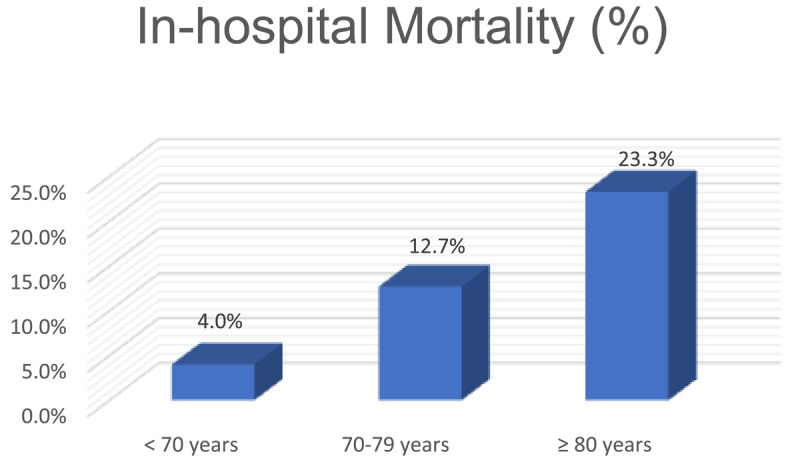
Comparison of the incidence of in-hospital mortality between the three groups.

In-hospital mortality of the three groups is shown in Figure 1. In-hospital mortality was significantly higher in the elderly (≥70 years) compared to the younger (<70 years) population (14.1% vs. 4%, p < 0.001). On subgroup analysis, it was higher in the octogenarians than in the septuagenarians (23.3% vs. 12.21%, p = 0.005).

#### Secondary outcomes

In-hospital complications of the elderly and younger cohorts are shown in Table 2A. Compared to the younger cohort, contrast-induced nephropathy occurred more frequently in the elderly (9.6% vs. 3.3%, p < 0.001) and TIMI major bleeding (1.7% vs. 0.9%, p = 0.027).

**Table 2A:** Comparison of in-hospital complications and outcomes between elderly and the younger populations.


VARIABLES	AGE GROUP ≥70 YEARS (N= 575)	AGE GROUP <70 YEARS (N= 3052)	*P* VALUE

Contrast-induced nephropathy	55 (9.6%)	100 (3.3%)	**<0.001**

Stroke	3 (0.5%)	9 (0.3%)	0.581

TIMI major bleeding	10 (1.7%)	26 (0.9%)	**0.027**

• Vascular access-related	2 (20% of major bleeding)	2 (7.7% of major bleeding)	

• Non-Vascular access-related	8 (80% of major bleeding)	24 (92.3% of major bleeding)	

Intracranial hemorrhage	4 (0.7%)	8 (0.3%)	0.092

Acute and subacute Stent thrombosis	6 (1.0%)	13 (0.4%)	**0.036**

In-hospital mortality	81 (14.1%)	122 (4%)	**<0.001**


In-hospital complications of Octogenarians and Septuagenarians are shown in Table 2B.

**Table 2B:** Comparisons of in-hospital complications and outcomes between Octogenarians and Septuagenarians subgroups.


VARIABLES	OCTOGENARIANS (≥80 YEARS) (N = 103)	SEPTUAGENARIANS (70–79 YEARS) (N = 472)	*P*VALUE

Contrast-induced nephropathy	13 (12.6%)	42 (8.9%)	0.267

Stroke	1 (1%)	2 (0.4%)	0.149

TIMI major bleeding	2 (1.9%)	8 (1.7%)	0.696

Intracranial hemorrhage	1 (1%)	3 (0.6%)	0.629

Acute and subacute Stent thrombosis	2 (1.9%)	8 (1.7%)	0.777

In hospital mortality	24 (23.3%)	57 (12.1%)	**0.005**


### Factors associated with in-hospital mortality

After adjusting for demographic and clinical variables, multivariate analysis was performed to identify factors associated with in-hospital mortality among elderly patients (Table 3A) and among the different age groups (Table 3B) undergoing primary PCI. Among the factors studied, Killip class ≥II was significantly associated with the risk of in-hospital mortality in all age groups. However, diabetes mellitus and chronic kidney disease were significantly associated with in-hospital mortality in the younger cohort only. On the other hand, Age ≥ 80 was only associated with in-hospital mortality in the elderly cohort.

**Table 3A:** Table 3A Predictors of in-hospital mortality among the Elderly cohort (multivariate analysis).


VARIABLES	OR (95% CI)	P VALUE

Sex	0.915 (0.540–1.549)	0.740

Age ≥ 80 years	2.317 (1.303–4.120)	**0.004**

Diabetes Mellitus	1.608 (0.947–2.730)	0.079

Hypertension	1.550 (0.902–2.664)	0.113

CKD	0.991 (0.592–1.659)	0.971

History of previous revascularization	0.284 (0.062–1.297)	0.104

Killip class ≥II at presentation	4.568 (2.760–7.560)	**<0.001**


**Table 3B:** Factors associated with in-hospital mortality stratified by age groups (multivariate analysis).


VARIABLES	OCTOGENARIANS (≥80 YEARS) (n = 103)		SEPTUAGENARIANS (70–79 YEARS) (n = 472)		AGE GROUP <70 YEARS (n = 3052)	

	OR (95% CI)	P value	OR (95% CI)	P value	OR (95% CI)	P value

Sex	0.86 (0.269–2.79)	0.812	1.6 (0.75–3.3)	0.26	1.42 (0.8–2.4)	0.635

Diabetes Mellitus	2.3 (0.820–6.73)	0.112	0.64 (0.33–1.4)	0.17	1.87 (1.26–2.93)	**0.004**

Hypertension	1.1 (0.372–3.230)	0.868	0223–0.843	0.14	0.94 (0.6–14)	0.790

Current smoker	1.6 (0.531–5.33)	0.373	0.370–1.272	0.231	0.87 (0.5–1.4)	0.598

History of previous revascularization	0.86 (0.297–45)	0.312	0.582–35.503	0.149	1.7 (0.7–40)	0.234

Killip class ≥II at presentation	6.57 (2.19–19.7)	**0.001**	0.136–0.441	**0.001**	7.4 (5–10.9)	**<0.001**

Chronic kidney disease	0.52 (0.181–1.56)	0.251	0.580–1.979	0.826	2.6 (1.6–4.1)	**<0.001**


### TIMI major bleeding in elderly

Only femoral access was significantly associated with TIMI major bleeding among the elderly. Female gender, octogenarian age, hypertension, diabetes mellitus, chronic kidney disease, and the use of Glycoprotein IIb/IIIa inhibitors had no significant association with TIMI major bleeding in univariate analysis (Table 4).

**Table 4:** Factors associated with TIMI major bleeding in the elderly (univariate analysis).


VARIABLES	TIMI MAJOR BLEEDING (N = 10)	NO TIMI MAJOR BLEEDING (N = 565)	*P* VALUE

Age ≥ 80 years	2 (20%)	101 (17.9%)	0.560

Female gender	4 (40%)	193 (34.2%)	0.446

Hypertension	7 (70%)	325 (57.5%)	0.539

Diabetes Mellitus	6 (60%)	271 (48%)	0.541

CKD	4 (40%)	212 (37.5%)	0.556

Glycoprotein IIb/IIIa inhibitors	4 (40%)	87 (15.4%)	0.139

Femoral access	9 (90%)	217 (38.4%)	**0.001**


## Discussion

While the elderly population faces an increased risk of cardiovascular disease, clinical trials often overlook older patients or only target those with lower risk profiles. Acute coronary syndrome (ACS) is responsible for approximately one-third of annual mortality among the elderly population in the United States (4). The present study focuses on a cohort of elderly individuals who underwent pPCI at our center, a main pPCI referral center in a 250-mile radius, serving around 2,000,000 people in upper Egypt (5). For this study, we defined “elderly” as 70 years or older, recognizing that the definition varies (6, 7). This cutoff was based on the observed age-related comorbidities and distinct healthcare needs within our specific patient population.

The main findings of the study indicate that the older age group had more comorbidities and worse clinical presentation, with a significantly higher frequency of complications and in-hospital mortality, particularly in the octogenarian group.

Our study included 3,627 patients who underwent pPCI, out of whom 575 (15.9%) were 70 or older. Only 2.8% (103 patients) were octogenarian, a lower percentage than other registries. We observed that the proportion of females was significantly higher in the elderly group than in the younger age group (34.3% vs. 18.3%, p = <0.001), consistent with previous studies (8,9,10,11,12,13).

Compared to other registries, the proportion of elderly patients with STEMI is lower. On average, other registries had 30% of elderly patients with STEMI (14, 15) and reached 39.6% in a Japanese nationwide registry. Notably, only 62.2 % of the elderly with STEMI in this registry received pPCI (16).

Octogenarians were less represented in our cohort, whereas compared to other studies, Octogenarians accounted for 6.2% and 6.7% (13). This might be explained by their propensity to exhibit less typical symptoms, which can lead to misdiagnosis and non-referral to our center. On the other hand, their tendency to present with a more severe/complicated clinical and/or hemodynamic presentation might have resulted in higher pre-referral mortality. Moreover, it should be noted that the mean age of the population and life expectancy in Africa is substantially lower than that in Europe and America (10,11,12, 15).

Elderly patients had a higher prevalence of hypertension in our cohort (57.7% vs. 40.9%, p < 0.001), which is consistent with the findings of Hafiz et al. (68.9% vs. 50.9%, P < 0.001) (17). There was no significant difference in diabetes mellitus between the major age groups in our study (48.2% vs. 44.3%, p = 0.171); this balance probably reflects increased mortality amongst those with diabetes mellitus and STEMI earlier in life (18). Furthermore, in the Japanese nationwide registry, diabetes was more common in patients younger than 75 years (16). Similarly, no significant increase in diabetes incidence was found among the elderly population (20.8% vs. 19.3%, p = 0.738) in the study conducted by Hafiz et al. (17).

In our cohort, CKD was almost four times more frequent in the elderly compared to younger patients. It has been demonstrated that the prevalence of CKD increases steadily with age, from 13.7% in the group aged 30 to 40, to 27.9% in patients aged over 70 (19). It is well established that any degree of renal insufficiency can increase cardiovascular morbidity and mortality (20,21,22).

The elderly were more likely to have worse clinical presentation compared to younger patients. The prevalence of Killip class II or worse was significantly higher in elderly patients compared to their younger counterparts (28% vs. 15.3%, p < 0.001). Cardiogenic shock incidence was significantly higher among the elderly population (11.7% vs. 5.1%, P < 0.001). This could be attributed to a higher prevalence of co-morbidities, delayed presentation (total ischemic time is 6.4 hours in the elderly vs. 5.8 hours in the younger cohort, P = 0.007), and worse angiographic findings than younger patients. This agrees with other studies (23,24,25) showing that STEMI patients presenting with cardiogenic shock are older and more likely to be female, have diabetes, a history of heart failure, previous myocardial infarction, and anterior myocardial infarction (24). In our study, compared to the septuagenarian patients, the octogenarians did not show any significant difference in risk factors or clinical presentation except for a significantly higher prevalence of CKD at presentation (55.3% vs. 34.11%, p = <0.001).

The angiographic data revealed that the elderly had a significantly higher incidence of the RCA being the culprit vessel. The association of other non-culprit vessel severe lesions was significantly higher in the elderly (40.3% vs. 30.7%, P ≥ 0.001). Moreover, there was a significantly higher prevalence of associated non-culprit severe LM disease in the elderly cohort (5.7% vs. 3.1%, P = 0.001) .

### Primary outcome

The present study showed a notable disparity in in-hospital mortality based on age group. Those aged 70 or older had more than triple the mortality of those under 70 (14.1% vs. 4%, p < 0.001). Within the entire older cohort, age itself remained a powerful predictor, with octogenarians (≥80 years) facing nearly double the mortality risk of septuagenarians (70–79 years) (23.3% vs. 12.21%; p = 0.005). Killip class II or worse was found to be an independent predictor of increased hospital mortality across all age groups in our cohort of patients. Chronic kidney disease and diabetes were independent predictors of in-hospital mortality in patients aged <70 years but not in the elderly. Among the octogenarians, Killip class II or worse was the only independent predictor of mortality.

Compared to other registries, octogenarians in our cohort have a higher in-hospital mortality (23.3%). In a previous STEMI database, those over 80 had an in-hospital mortality rate of 14.4% vs. 3.6% in those under 80 (26). However, the median time from chest pain onset to presentation in the octogenarians and septuagenarians was 99 minutes and 88 minutes, respectively (26). While in our study, the total ischemic time in the octogenarians and septuagenarians was 7 and 6 hours, respectively.

In concordance with our study, Perl et al. found that octogenarians had a higher rate of all causes of death at one month compared to the septuagenarians (19.0% vs. 12.3%) based on a database from two Mediterranean medical centers (6). This was also shown in another study that reported a 30-day mortality of 29.7% in octogenarians (13), where the increased mortality was attributed to more severe clinical presentation and long door-to-balloon time (1.4 hours).

The increased in-hospital mortality of septuagenarians and octogenarians in our study could be attributed to the atypical presentation, severe clinical presentation in the form of Killip class ≥II and cardiogenic shock, long total ischemic time as well as the increased number of associated non-culprit vessel severe lesions (40.3% vs. 30.7%, p < 0.001).

It is imperative to acknowledge that although the elderly population has a higher mortality rate, pPCI still significantly reduces the mortality rate within the first 24 hours for all genders and age groups (27). STEMI mortality has declined in the past few decades (28). The in-hospital mortality rate for pPCI is currently around 5.9% (29). Nonetheless, age is an independent predictor of mortality in older patients and remains one of the main predictors of outcomes in STEMI and ACS in general (30). Previous studies have shown that mortality rates of acute myocardial infarction increase exponentially after the age of 65. For patients aged 65–75, the rate is 7.1%, while for those over 75, it is 11.1% (31). Hafiz et al. (32) reported a high in-hospital mortality rate of 15.5% for pPCI in patients aged ≥75, compared to 2.7% in patients <75. Age, diabetes, and renal failure were associated with higher mortality (32).

### Secondary outcomes

In our study, elderly patients suffered from more in-hospital complications compared to younger patients. However, the octogenarian group did not show significant differences in complications compared to Septuagenarians.

#### 1. Contrast-induced nephropathy

Following pPCI, we observed a significantly higher incidence of CIN in patients aged 70 years or older than in younger patients (9.6% vs. 3.3%, p = <0.001). This supports the previous findings of a high incidence of CIN in the elderly, particularly in emergency/urgent procedures compared to elective procedures. In a recent study, CIN occurred in 26.7% of very elderly patients (mean age 80 years) undergoing PCI for ACS and was more frequent in STEMI than NSTEMI (33). However, it is important to highlight that the prevalence of CKD at presentation in our study was significantly higher in the elderly than their younger counterpart.

#### 2. Bleeding

The incidence of TIMI major bleeding was higher in patients aged ≥70 years than in younger patients (1.7% vs. 0.9%, p = 0.027). Our findings agree with previous studies where elderly patients had a higher risk of bleeding following PCI due to the high prevalence of co-morbidities, particularly hypertension and renal impairment (34). In a previous study that included patients older than 75, all bleeding occurred in 15.4% of the entire cohort within 30 days after PCI (35).

Different observational studies have demonstrated bleeding as an independent predictor of mortality following PCI (36, 37). Bleeding has a greater impact on prognosis in the elderly due to increased arterial stiffness, endothelial dysfunction, and more frequent LV dysfunction (38).

The performance of different risk scores in predicting bleeding in the elderly is notably less robust than in younger patients (39). Old age, female gender, renal impairment, and history of bleeding impairment are well-established risk factors for bleeding (40). In our study, we conducted a univariate analysis and found that only femoral access was significantly correlated with TIMI major bleeding in elderly patients. Despite the potential technical challenges in the elderly, several studies have shown that radial access reduces bleeding risk and improves outcomes (41,42,43,44). Among our cohort, younger patients underwent radial access more often than the elderly (70.2% vs. 60.7%, respectively, p < 0.001). The limited number of TIMI major bleeding events in the different age groups in the present study restricted the ability to conduct robust subgroup analyses and make definitive conclusions about age-specific bleeding risks.

#### 3. Acute and subacute stent thrombosis

In our study, acute and subacute stent thrombosis incidence was significantly higher in the elderly compared to those under 70 years old (1.0% vs. 0.4%, p = .036). The relation of age to stent thrombosis has been controversial. Raposeiras-Roubin et al. (41) found that age was a significant independent predictor of stent thrombosis at one month and one year after the procedure. Notably, this study also identified impaired renal function as an independent predictor of stent thrombosis. Kumar et al. (42) also found that older age is associated with increased stent thrombosis, but not as an independent predictor.

On the other hand, Ohno et al. (43) found younger age to be an independent predictor of stent thrombosis (45). It is important to highlight that the last two studies relied on data from patients who presented with stent thrombosis rather than follow-up data following pPCI, which may cause survival bias.

## Limitations

The study’s limitations include reliance on data from a single observational cohort primarily focusing on in-hospital outcomes and a lack of long-term follow-up data. The study does not include data on alternative treatment strategies, such as fibrinolysis or patients who have declined invasive therapy and/or have not been referred to our center. Data on admission LVEF is not available. The event rates for some adverse events among the different age groups were relatively low, which limited the statistical power to draw definitive conclusions about these outcomes.

## Conclusions

The main findings of the study indicate that the older age group had more comorbidities and worse clinical presentations, with significantly higher frequency of complications and in-hospital mortality, particularly in the octogenarian group. Nevertheless, pPCI is still the standard of care in STEMI, with no known age limit. Given the atypical and delayed presentation it often experiences, this age group needs a high index of suspicion. Timely pPCI with strict measures to reduce the risk of contrast-induced nephropathy and bleeding may further improve the outcomes.

## Data Accessibility Statement

Availability of data and material: The datasets used and/or analyzed during the current study are available from the corresponding author upon reasonable request.
